# Nursing

**Published:** 1876-02

**Authors:** 


### NURSING.

A GOOD nurse is better than physic. That nurse should be a woman: her soft hand, her soothing voice, her sympathetic nature, her capabilities of endurance, her alertness, her tidiness, and scrupulous cleanliness, make her incalculably better adapted for attending on the sick, than man possibly can be. Many a valuable life would be saved, if nursing could be made a "calling" for women; if they could be regularly instructed in the performance of duties of that character. If we were ill of any disease known to man, especially scarlet fever and small-pox, we would rather take our chance for life with a good woman-nurse than any apothecary shop.

Let us give an illustration which will impress the truth on the mind indelibly, that it is a greater calamity, in sickness, to be without a good nurse, than to be without a doctor. That human angel, FLORENCE NIGHTINGALE, says, that during the first six months of the Crimean campaign, the deaths among the soldiers were at the rate of sixty out of a hundred, per annum; during the last six months, with the same physicians, the mortality among the *sick* was only two-thirds of what it was among the *healthy* soldiers in England—this great change arose from improvements carried out by the Sanitary Commissioners, in reference to the care of the sick and their surroundings.

Sixty persons dying out of a hundred in a year, is worse than the great plague in London, worse than the terrible ravages of cholera.

Ordinarily, only twenty-one persons out of every thousand die in a year. It may be instructive to know what were some of the circumstances, the removal of which makes such a remarkable change. Each patient had only one-fourth of the room necessary; the windows were not hoisted so as to admit fresh air; the ground about the buildings was always wet, for want of draining; dead animals lay around on the ground for several weeks; the floors were incrusted with dirt, and could not be washed, for the necessary scrubbing caused the rotten planks to crumble; the walls and ceilings were saturated with decomposing matters; rats and vermin swarmed everywhere, and to exhibit the want of furniture, utensils, &c., a common bottle was used as a candlestick

Not much better, according to a correspondent of the *Boston Medical Surgical Journal*, was the condition of things in the building where originated the disease which destroyed so many people a year ago—a water-closet under the same roof was so full, that the filth came up through the cracks of the floor, when he stepped on it; and other things to match.

Physic has power, sometimes almost miraculous; but it is shorn of its locks, and is weak as an infant when it has to contend against dirt, dampness, and the want of fresh and pure air, nor can the best nurses in the world do better under the circumstances. Let everything about the sick be perfectly dry, and scrupulously clean, with an orderly arrangement of everything about the bed and room; be quiet in movement, cheerful in countenance, prompt in action, with a plenty of pure air steadily circulating. Have a large, high room, with windows facing the sun, for a greater part of the day; let the fire-place be always open; remove all bottles and other "signs" of physic; allow no standing liquids, not even pure water; and have no hanging garments about. As you love the invalid, attend to these things.

